# miR-145-5p/Nurr1/TNF-α Signaling-Induced Microglia Activation Regulates Neuron Injury of Acute Cerebral Ischemic/Reperfusion in Rats

**DOI:** 10.3389/fnmol.2017.00383

**Published:** 2017-11-21

**Authors:** Xuemei Xie, Li Peng, Jin Zhu, Yang Zhou, Lingyu Li, Yanlin Chen, Shanshan Yu, Yong Zhao

**Affiliations:** ^1^Department of Pathology, Chongqing Medical University, Chongqing, China; ^2^Key Laboratory of Neurobiology, Chongqing Medical University, Chongqing, China

**Keywords:** Mir-145-5p, Nurr1, TNF-α, cerebral, ischemic/reperfusion

## Abstract

Nurr1 is a member of the nuclear receptor 4 family of orphan nuclear receptors that is decreased in inflammatory responses and leads to neurons death in Parkinson’s disease. Abnormal expression of Nurr1 have been attributed to various signaling pathways, but little is known about microRNAs (miRNAs) regulation of Nurr1 in ischemia/reperfusion injury. To investigate the post transcriptional regulatory networks of Nurr1, we used a miRNA screening approach and identified miR-145-5p as a putative regulator of Nurr1. By using computer predictions, we identified and confirmed a miRNA recognition element in the 3′UTR of Nurr1 that was responsible for miR-145-5p-mediated suppression. We next demonstrated that overexpression of Nurr1 inhibited TNF-α expression in microglia by trans-repression and finally attenuated ischemia/reperfusion-induced inflammatory and cytotoxic response of neurons. Results of further *in vivo* study revealed that anti-miR-145-5p administration brought about increasing expression of Nurr1 and reduction of infarct volume in acute cerebral ischemia. Administration of anti-miR-145-5p promotes neurological outcome of rats post MCAO/R. It might be an effective therapeutic strategy to relieve neurons injury upon ischemia/reperfusion of rats through interrupting the axis signaling of miR-145-5p- Nurr1-TNF-α in acute phase.

## Introduction

Cerebral ischemia/reperfusion (I/R) injury-induced neuronal cell death is the most difficult problem to solve in clinical stroke (Al-Mufti et al., 2017). Activated microglia have been reported to act as sensors that detect abnormal metabolic changes following I/R, including reactive oxygen species and inflammatory cytokines (Yuan et al., 2014; Fumagalli et al., 2015). Microglia can release excessive proinflammatory cytokines and/or cytotoxic factors, such as tumor necrosis factor-α (TNF-α), interleukin-1β (IL-1β) and nitric oxide (NO), which have been shown to contribute to neuronal damage (Yuan et al., 2014; Roqué et al., 2016; Gullo et al., 2017). Therefore, suppressing overreaction of the microglial inflammatory response may be an efficacious therapeutic strategy to alleviate progression of stroke.

Nurr1 (NR4A2) is a member of the nuclear receptor 4 family of orphan nuclear receptors (Kim et al., 2015; Zou et al., 2017), and has been studied extensively in Parkinson’s disease recently. Nurr1 has been shown to inhibit expression of proinflammatory neurotoxic mediators by docking to NF-κB-p65 on target inflammatory gene promoters in microglia (Saijo et al., 2009; Kim et al., 2015). Contra-directional coupling of Nur77 and Nurr1 are involved in neurodegeneration or injury by regulating endoplasmic reticulum stress and mitochondrial impairment (Gao et al., 2016; Wei et al., 2016). Cystatin C induces VEGF expression and attenuates PC12 cell degeneration by regulating p-PKC-α/p-ERK1/2-NURR1 signaling and inducing autophagy in Parkinson’s disease (Zou et al., 2017). Taken together, these findings indicate that Nurr1 could be a promising therapeutic target in neuronal diseases. However, the specific functions and/or mechanisms of Nurr1 in I/R injury are still unknown.

Increasing recent evidence suggests important roles for microRNAs (miRNAs) in molecular processes of cerebral ischemia pathogenesis, which involve fast post-transcriptional effects and simultaneous regulation of various target genes (Ouyang et al., 2015; Minhas et al., 2017). Although several miRNAs have been reported to target Nurr1 in cancer cells (Yang et al., 2012; Wu et al., 2015; Beard et al., 2016), interaction between miRNAs and Nurr1 in cerebral I/R injury is still poorly understood.

In the present study, we therefore sought to determine: (1) how Nurr1 level and Nurr1-related microRNAs change in the brain of MCAO/R rats, an *in vivo* model; (2) in *in vitro* studies, what are the post-transcriptional regulatory network of Nurr1 and the specific mechanisms of Nurr1 on microglia activation; (3) in an *in vivo* study, whether miR-145-5p/Nurr1/TNF-α signal exerts neuron injury function upon cerebral ischemia-reperfusion in rats.

## Materials and Methods

### Experimental Animals and Ethics Statement

About 170 adult male Sprague-Dawley (SD) rats (weight between 250–300 g, 60–80 d) were purchased from the Laboratory Animal Centre of Chongqing Medical University used for the *in vivo* study. Brain tissues from newborn SD rats (0–24 h) were used to culture primary neurons of cerebral cortex and glia cells. This study has been carried out within an appropriate ethical framework. All experimental procedures were performed in accordance with the National Institutes of Health (NIH) Guide for the Care and Use of Laboratory Animals and approved by Biomedical Ethics Committee of Chongqing Medical University. Maximum efforts have been made to minimize the number of animals used and their suffering. Adult male Sprague-Dawley rats were used that is exempted from ethics approval.

### Cell Culture of Primary Microglia and Neurons and OGD/R Treatment

Primary glial cells were isolated from the cerebrum and cerebellum of rats (1-day-old) and placed in 6-well plates at a density of 1.2 × 10^6^ cells/ml of DMEM supplemented with 10% fetal bovine serum (FBS), non-essential amino acids, and insulin. Plates were then placed in a humidified incubator with 5% CO_2_/balanced with air (result: 20% O_2_) at 37°C with medium change per 48 h. Microglia were isolated from the mixed glial population after confluent (about 2 weeks) by a method previously described (Jose et al., 2014). Microglia cultures with more than 96% purity were used for the study.

Primary neurons were obtained from the cerebral cortex of 1 day-old rats and cultured as described in our previous studies (Zhou et al., 2015; Chen et al., 2016). Approximately 2.0 × 10^6^ cells per 2 mL of Neurobasal Medium containing 1% penicillin (Pen, 100 U/mL) and 1% streptomycin (Strep, 100 U/mL), and 2% B27 supplement were seeded per well. Neurons were cultured in a humidified incubator with 5% CO_2_ at 37°C. After 6–7 days of culture *in vitro*, cells were examined to ensure more than 90% purity of neurons which could be used for further study.

The thorough method of OGD/R was conducted as previously described (Tauskela et al., 2003). Briefly, microglia/neurons were washed and cultured with glucose-free DMEM, which had been previously equilibrated with 1% O_2_ + 5% CO_2_ + 94% N_2_ at 37°C for 2.0 h in an incubator. Cells were exposed to hypoxia by placing them in an incubator filled with a gas mixture of 1% O_2_ + 5% CO_2_ + 94% N_2_ for 1.5 h at 37°C. The glucose-free DMEM were then changed back to their special mediums and cultures were returned to the normal incubator for recovery times of 0.5 h, 1.0 h, 2 h, 6 h or 12.0 h. An appropriate time of reoxygenation was selected for subsequent studies. Cells exposed to normoxia were used as negative control.

### Coculture of Microglia and Neurons

Primary microglia were seeded onto Transwell permeable support membrane inserts (Corning, NY, USA) at a density 1.0 × 10^6^/well in DMEM medium supplemented with 10% FBS and allowed to settle and grow for 24 h, which constituted the upper chamber in a 2-chambered microglia-neuronal cell coculture system. The cultured neurons were seeded into the bottom of 6-well plates at a density of 2.0 × 10^6^ cells/well in specific Neurobasal medium as described above until confluence at 24 h. These neurons were then cocultured with microglia-containing inserts for 48 h in the following conFigureuration: (1) neurons incubated with empty inserts lacking microglia (neurons only); (2) neurons incubated with inserts containing microglia that had not been exposed to OGD/R (microglia normoxia (nor.) + neurons); and (3) neurons incubated with inserts containing activated microglia that had been previously exposed to OGD/R for 2 h (microglia OGD/R 2 h + neurons). Following coculture, the two coculture chambers were disassembled, and exposed neurons were washed with PBS and harvested for micro-RNA, mRNA, and/or cell viability analysis (Chen et al., 2012, 2016).

### Construction of Middle Cerebral Artery Occlusion and Reperfusion (MCAO/R) Model for *in Vivo* Experiments

SD rats were fed and housed under standard conditions before operation, and the room temperature was monitored at 24–28°C throughout the surgical procedure. MCAO model of rats were construct by method of intraluminal vascular occlusion as previously described in our laboratory (Chen et al., 2012, 2016). All the surgical procedures were performed successfully under anesthesia with 3.5% chloral hydrate (350 mg/Kg, intraperitoneal injection). The nylon filament with its tip rounded (diameter 0.24–0.28 mm), determined by the animal weight, was inserted into the middle cerebral artery for 1.5 h. Reperfusion of the ischemic artery was established by withdrawal of the filament until the tip cleared the lumen of the external carotid artery. Regional cerebral blood flow was monitored by an ultrasonic blood flow meter during the operation.

A successful occlusion and reperfusion of MCAO model was evaluated by methods of Neurological Outcome Assays and infarction volume assays as described in references (Lourbopoulos et al., 2008; Wang et al., 2014). Rats of sham-operated were subjected to the same surgical procedures as MCAO/R group except for occlusion of the external carotid artery. Animals which had blood reperfusion below 70% or died during reperfusion were excluded from analysis.

After MCAO, the experimental rats in each group (*n* = 5) were euthanized randomly at the end of reperfusion for 2, 6, 12, 24 and 48 h to detect the alterations of various index. SD rats were divided randomly into 15 groups. Before suffered MCAO/R, the groups were: null group, scramble group, anti-miR-145-5p group, miR-145-5p mimic group, Nurr1-siRNA group, Nurr1 activation plasmid group (Nurr1; intra-cerebroventricular injection). After MCAO/R, the groups were: sham-operated (sham) group, MCAO/R group, null + MCAO/R group, scramble + MCAO/R group, anti-miR-145-5p + MCAO/R group, miR-145-5p mimic + MCAO/R group, Nurr1-siRNA + MCAO/R group, Nurr1 + MCAO/R group. miR-145-5p mimic and anti-miR-145-5p were administered to the animals before they underwent any surgery via intracerebroventricular infusion.

### Neurological Outcome Assays

Two different assays were used to assess the neurological deficit of the rat on days 1, 7 and 14 after MCAO/R after the induction of stroke. A modified Neurological Severity Score (mNSS) was applied, three motor tests and two sensory tests were included, which are evaluated by total score 18 (Lourbopoulos et al., 2008; Wang et al., 2014). The scores are added up to a score between 12 (severe impairment) and 6 (no neurological impairment). Additionally, the foot fault test was performed to assay fine motor skills and proprioception. The amount of foot faults was computed via ((number of left forelimb faults) + (number of left hindlimb faults))/((total number of left forelimb steps) + (total number of left hindlimb steps)) (Lourbopoulos et al., 2008; Wang et al., 2014).

### Infarct Volume Analysis

SD rats were sacrificed upon different treatment, and the brain tissue was prepared into 3 mm sections for 2,3,5-triphenyltetrazolium chloride (TTC) staining (Sigma-Aldrich, St. Louis, MO, USA). Cerebral tissue sections were incubated with 2% TCC solution in the dark room at 37°C for 5 min; the tissue sections were then fixed in 4% paraformaldehyde. The living brain tissues was bright red, however the ischemic or necrotic tissues were pale. The digital images were further analyzed by Sigma Scan Pro 5.0 Software. The real infarct volume (cortex, striatum and hemisphere), after excluding edema, was calculated by subtraction of the ipsilateral non-infarcted regional volume from the contralateral regional volume. The real infarct volume was then divided by the contralateral regional volume as a percent of the contralateral region (McCarter et al., 2017).

### RNA Extraction, Reverse Transcription and RT-qPCR

Total RNA and miRNA in the brain tissues or primary cultured cells were extracted using TRIzol^®^ with miRcute miRNA isolation kits (Tiangen Biotech, Beijing, China). The concentration and integrity of sample RNA were determined using Nanodrop ND-1000 spectrophotometry (Nanodrop Tech, Rockland, DE, USA) and denaturing agarose gel electrophoresis. The cDNA was obtained by reverse transcription with an miRcute miRNA First-Strand cDNA Synthesis kit (Tiangen Biotech, Beijing, China). The RT-qPCR was performed using iQ5 RT-qPCR detection system (Bio-Rad Laboratories, Inc., Hercules, CA, USA).

Quantitation of Nurr1 and TNF-α mRNAs was performed using SYBR green assay (Zhou et al., 2015). Specific primer sequences were designed and generated by Sangon Biotech (Shanghai, China). For miRNA detection, the cDNA was obtained by reverse transcription with an miRcute miRNA First-Strand cDNA Synthesis kit (Tiangen Biotech, Beijing, China). Stem-loop qRT-PCR reactions were performed according to manufacturer’s protocols using miRNA specific stem-loop primers (Sangon Biotech, Shanghai, Co., Ltd.). Real-Time PCR reactions were conducted on a PCR amplifier (CFX-96 Content Real-time System). The endogenous control of both mRNAs and miRNAs was Ribosomal RNA (18S rRNA) for the quantitative PCR (qPCR) assays because it is known to expressed stably in cerebral ischemic conditions (Sepramaniam et al., 2010; Liu F. J. et al., 2015).

### miRNA Profiling

A miRNA microarray was performed according to the MicroRNA Expression Profiling Assay Guide (Illumina Inc., San Diego, CA, USA). The extracted RNA from the cerebral cortex of rats was labeled with Hy3 dye at 3′-end using the miRCURY LNA Power Labeling Kit (Illumina Inc.) in the 500 ng intact total RNA sample (Liu C. et al., 2015). The labeled miRNAs were hybridized to the BeadChip for 16–18 h, according to manufacturer’s instructions (Illumina Inc.). The microarray chips were then washed and scanned by InnoScan700, microarray scanner and analyzed by GenomeStudio™ Gene Expression Module v1.0 software (Illumina Inc.). The *p* value < 0.01 was considered to be accurately detected and selected for further analysis.

### miRNA Target Prediction and Mutagenesis

Six miRNA target-prediction algorithms were used to identify putative miRNA regulators of Nurr1: http://www.mirbase.org/; http://mirtar.mbc.nctu.edu.tw/human/index.php; http://www. targetscan.org; http://www.microrna.org; http://mirdb.org/cgi-bin/search.cgi; and http://pictar.mdc-berlin.de/. By using these algorithms, a putative seed region was determined and mutated using site-directed mutagenesis (Mutagenex, Inc., Piscataway, NJ, USA; Jeyaseelan et al., 2008). Reporter constructs containing either the wild-type or mutated 3′UTR were used to demonstrate miR-145-5p specificity in the Nurr1 3′UTR.

### Cloning of Nurr1 3′UTR and Dual Luciferase Reporter Assay

The 3′UTR of *Nurr1* was amplified by PCR using gene specific primers and cloned into firefly-luciferase-expressing vector pMIR-REPORT (Ambion, Austin, TX, USA). HEK293T cell was used in this study for its high transfection efficiency (>90%; Karra and Dahm, 2010). Briefly, HEK293T cells were cultured in 24-well plates and transfected with 40 nM anti- or mimic microRNAs for 3 h followed by 200 ng/well of pMIR-REPORT constructs for 3–5 h. Before lysed for measurement of luciferase activity, cells were then left to recover in CO_2_ incubator at 37°C for 48 h. The effect of miRNA binding with 3′UTR of Nurr1 quantified using Dual luciferase assay according to manufacturer’s protocol (Cat # E1910; Promega, USA). Transfection efficiencies were normalized to those of cells by co-transfecting with the Renilla-luc-expressing vector pRL-CMV (Cat # E2261; Promega, Madison, WI, USA) at 5 ng/well.

### Gene Transfection and siRNA Interference Experiments *in Vivo* and *in Vitro*

The pcDNA-Nurr1 overexpression lentivirus was constructed and packaged by Neuron Biotech (Shanghai, China). The Nurr1-siRNA, scramble siRNA and siRNA reagent system were purchased from Santa Cruz Biotechnology Inc. (Cat # sc-36111). For *in vitro* assays, the confluent microglia and neuron were transfected with pcDNA- Nurr1 using Lipofectamine 2000 (Invitrogen, Carlsbad, CA, USA) according to the manufacturer’s instructions. The siRNA interference of Nurr1 was performed according to the manufacturer’s instructions. Cells transfected with empty vectors (EV) and un-transfected cells (Null) served as negative control groups. Sustained Nurr1 overexpression or down-regulation were confirmed by qRT-PCR and western blot analysis 72 h after transfection. Scramble siRNA and Nurr1-siRNA were dissolved in RNase-free water to a final concentration of 2 μg/μL, and injected ipsilaterally into the left lateral cerebral ventricle 48 h before MCAO.

### Cell Viability Analysis

The cultured neurons with different treatments were grown in 96-well plates separately at a density of 2.0 × 10^5^ cells/mL and then harvested to analyze cell viability using The Cell Titer 96 Aqueous One Solution cell proliferation assay with 3-(4,5-dimethylthiazol-2-yl)-2,5-diphenyltetrazolium bromide (MTS; Cat # G3582, Promega, USA). Absorbance was measured at 490 nm using a microplate reader.

### Chromatin Immunoprecipitation (ChIP) Assay

ChIP assay were performed using EZChIP kit which contains all necessary reagents to perform 22 individual (ChIP) reactions using inexpensive protein G agarose beads (Cat # 17-371, EZ-ChIP™, Merck Millipore, USA). Briefly, for each experimental condition, 2 × 10^7^ rat primary microglia were needed. Cells were cross-linked for 10 min with 1% formaldehyde. Samples were incubated with Anti-Nurr1 antibody [N1404]—ChIP Grade (ab41917; Cat # ab41917, 1:100, Abcam, Cambridge, MA, USA; 1:100 in ChIP dilution buffer - 0.01% SDS, 1.1% Triton X-100, 1.2 mM EDTA, 16.7 mM Tris-HCl (pH 8.1), 167 mM NaCl, proteanase inhibitor cocktail) for 16 h at 4°C. A 150-bp region of the rat proximal TNF-α promoter was amplified spanning the NF-κB site. Protein binding was detected using RT-qPCR.

### Immunofluorescence Staining

Fresh freeze brain sections (10 μm) were incubated with 10% normal goat serum/0.3% Triton-X 100 diluted in PBS blocking solution at 37°C for 1 h. Slides were then incubated with the following corresponding primary antibodies: Polyclonal rabbit anti-rat Nurr1 (1:100 dilution; sc-991, Santa Cruz, CA, USA) and monoclonal mouse anti-goat CD68 (1:50 dilution; Cat # ab1211, Abcam, Cambridge, MA, USA) overnight at 4°C. Following incubation, slides were then washed in 0.1 M PBS and incubated for 1 h with the following secondary antibodies: goat anti-rabbit Immunoglobulin G (1:100 dilution; zhongshanjinqiao, China) and goat anti-mouse Immunoglobulin G (1:200 dilution; zhongshanjinqiao, China). DAPI was used to stain the nuclei (Sigma-Aldrich). All the sections were visualized under a Nikon ECLIPSE Ti fluorescence microscope, loaded with a CoolSNAP photometrics camera at 400× magnification.

### Western Immunoblot Analysis

Cell lysis buffer of cultured neurons and the rat cortex supplemented with proteinase inhibitors were used to extract total protein. The quantified proteins were separated by 8% SDS-PAGE and transferred onto polyvinylidene fluoride (PVDF) membranes. The membranes were subsequently blocked with 5% skim-milk for 2.0 h at room temperature and incubated in primary antibody overnight at 4°C. Dilutions for primary antibodies were as follows: Polyclonal rabbit anti-Nurr1 and anti-TNF-α (1:500 dilution; 6945S, Cell Signaling Technology, USA), Polyclonal mouse anti-β-actin (1:5000, ABclonal, Wuhan, China, AC004) and anti-IL1β (1:1000, Bioworld technology, USA). The membranes were then incubated with appropriate secondary antibodies at 37°C for 2 h (dilution 1:5000, Sangon Biotech, Shanghai, Co., Ltd.). The density of bands was detected using an enhanced chemiluminescence system (Cat # 32132, Pierce™ ECL Plus Western Blotting Substrate ECL Plus, USA), and the gray value of bands was quantified using ImageJ analysis software. The relative expression quantity of protein was scored as the ratio of target protein intensity to β-actin staining intensity.

### Statistical Analysis

All data are expressed as mean ± SD. One-way analysis of variance (ANOVA) followed by Bonferroni test was used to compare results among all groups. The Spearman’s correlation test was used to examine the correlations of relative expression levels between protein and miRNA. The SPSS 17.0 software package was used to execute all statistics. All experiments were independently repeated at least three times. *p*-values < 0.05 were considered statistically significant.

## Results

### Nurr1 Expression Decreased in Both the Cerebral Cortex and Hippocampus of Rats after MCAO/R

**Figure 1:**
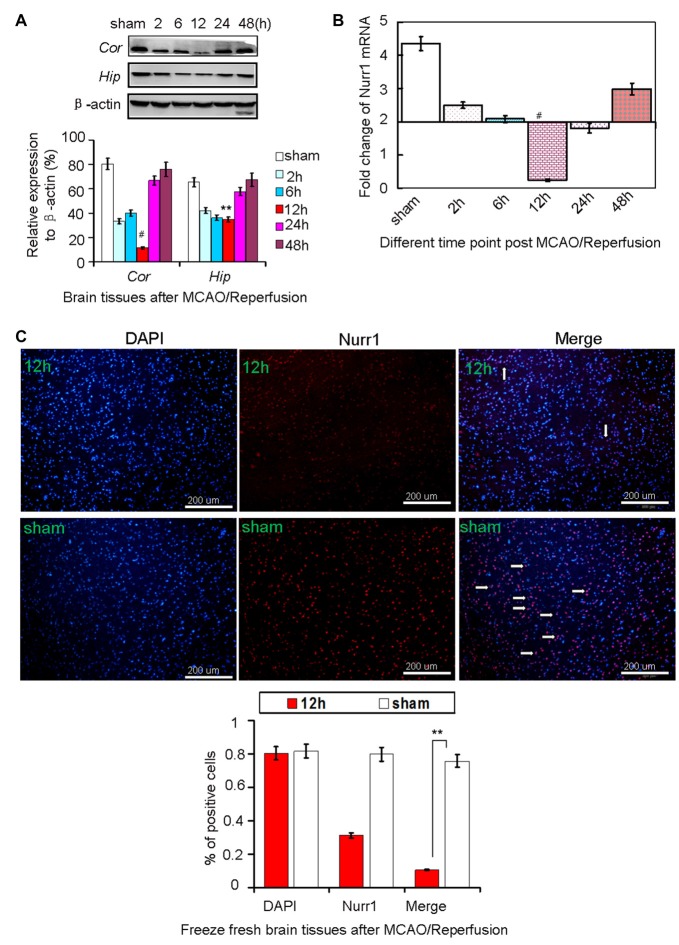
Nurr1 expression at 2, 6, 12, 24 and 48 h after MCAO/R. **(A)** Nurr1 protein was measured by Western blot at each time point upon cerebral ischemia/reperfusion. The gray intensities of the bands were quantified using ImageJ software and presented as percentage of β-actin (internal control, %). Nurr1 expression decreased sharply and reached a minimum at 12 h in cerebral cortex (*Cor*) and hippocampus (*Hip*) of rats. **(B)** Nurr1 mRNA was measured by RT-qPCR and presented as relative expression (mean ± SD, *n* = 3). **(C)** Nurr1 protein expression was significantly declined in the infarct side of cortex by immunofluorescence staining from fresh freeze brain sections 12 h after MCAO/R. **p < 0.01, ^#^*p* < 0.001.

**Figure 2:**
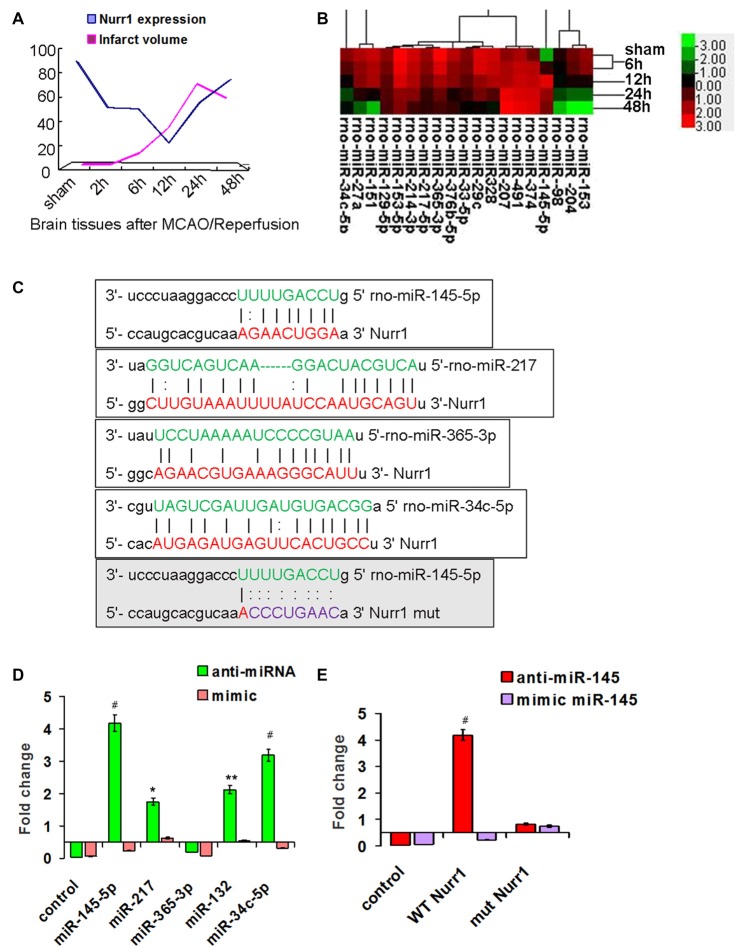
Screening for miRNAs that directly target the 3′UTR of Nurr1. **(A)** The infarct volume was negatively correlated with Nurr1 expression some extent by Pearson correlation test, but was not statistically significant (correlation coefficient −0.457, *P* = 0.362). **(B)** Heat map of predicted miRNAs expression in cerebral cortex tissue from sham and MCAO/R animals (one-way analysis of variance (ANOVA), *p* < 0.05). **(C)** The predicted binding sites of selected miRNAs (in green color) to the 3′UTR of Nurr1 (in red color) is mapped in this figure. Nucleotides which were altered for mutational studies are marked in gray color of background. **(D)** Quantitation of the effects of anti miRNAs and miRNA mimics interaction with the 3′UTR of Nurr1. **(E)** Quantitation of the effects of anti-miR-145-5p and miR-145-5p mimic interactions with the normal binding sites (WT) and mutated binding sites (mut) in 3′UTR of Nurr1. *p < 0.05, **p < 0.01, ^#^*p* < 0.001.

Every three rats were euthanized randomly in each group at different time point and different treatment after MCAO/R. Rats in the sham group were used as negative control. Approximately 138 rats were euthanized in the whole study, with the 82.4% (138/170) survival rate of the animals after MCAO surgery. Both mRNA and protein levels of Nurr1 decreased from the start of ischemic stroke, reached a minimum at 12 h, and then increased until 48 h (Figures 1A,B). Similarly, Nurr1 expression significantly decreased on the infarct side of the cortex and hippocampus of fresh frozen brain tissue sections as shown by immunofluroescence assays (Figure 1C). In addition, brain tissues were stained with 2,3,5-triphenyl-tetrazolium chloride (TTC) to measure infarct size on the ipsilateral side. Infarct size peaked at ~24 h and then decreased progressively until 48 h after occlusion (Figure 2A). Infarct volume was negatively correlated with Nurr1 expression in the cortex, but this was not statistically significant using the Pearson correlation test (correlation coefficient −0.457, *P* = 0.362).

### Screening for miRNAs that Directly Target the 3′UTR of Nurr1

miRNA expression was measured in our miRNA profiling analysis of rat cortex samples at different time point after MCAO/R (Figure 2B). Eleven miRNAs, including miR-145-5p, miR-34c-5p, miR-365-3p, miR-214-3p, miR-151, miR-27a, miR-153-5p, miR-365-3p, miR-33-5p, miR-217-5p and miR-129-5p, were differentially and significantly expressed (*P* < 0.05; Figure 2B). Among them, seven putative relevant miRNAs were screened by bioinformatic analysis of databases to identify miRNAs that regulate Nurr1 directly through its 3′UTR (Table 1). The predicted binding sites of the selected miRNAs are shown in Figure 2C.

**Table 1:** microRNAs that potentially regulate Nurr1 through its 3′UTR by bioinformatic analysis prediction in three foremost databases in this field.

miRNA	Mature sequence	Target Score of Nurr1 prediction			Other target genes
		MIRDB	TARGETSCAN (Pct)	MICRORNA (mirSVR)	
>rno-miR-145-5p	GUCCAGUUUUCCCAGGAAUCCCU	81	0.30	−1.03	SRGAP2, Abhd17c, Ebf3, Add3, Ythdf2, Kcna4, Elmo1, Snx8, Abhd17b, Cbfb, Ino80, Ap1g1, Rev3l, Scamp3, Mdfic, Spop, Zfyve9, Mpzl2, Prkx, Pikfyve, Angpt2, RGD1562865, Zbtb6, Cachd1, Zfhx4, Zfyve26, Dusp6, Actg1, Fam135a, H1f0, H2afx, Spsb4, Lox, Rapgef2, Gosr2, Coro2b, Snx27, Cdo1, Spats2, Nol9, Csmd3
>rno-miR-365-3p	UAAUGCCCCUAAAAAUCCUUAU	−	0.28	−0.55	Ing3, Fads1, Casp6, Usp48, Sgk1, Eltd1, Adm, Pax6, Ublcp1, Mrs2, Crbn, Gcom1, MGC112830, Ets1, Rgs1, Tcp11l2, Otc, Tyms, Pebp1, Inhbe, Larp1b, Igf1, Csk, Ubfd1, Dcun1d5, Prkab2, Acsm5, Plcb4, Cpox, Arrb2, Fgfbp1, Nat15, Mare, IL10, Grb7, Galt, Nphs1, Aldob
>rno-miR-214-3p	ACAGCAGGCACAGACAGGCAG	−	<0.1	−0.28	Zbtb20, Luzp1, Atp2a3, Camta1, Smarcd1, Naa15, Endod1, Sec24c, P2rx6, Ldoc1, Pla2g3, Cpsf4, Psmd11, Ammecr1l, Myo18a, Zfand3, Numa1, Ccdc167, Tspan9, Sema4d, Phf6, Zbtb39, Ppih, Itpk1, Atp11c, Pter, Slc8a1, Wnt2, Diaph1, Naa50, Qsox1, Nfatc2, Plekhj1, Ezh1, Hr, Ctdsp1, Fbln5, Nomo1, Slc45a4, Lhx6, Socs7
>rno-miR-33-5p	GUGCAUUGUAGUUGCAUUGCA	57	<0.1	−0.99	Abca1, Zfp281, Crot, Pdgfra, Ywhah, Hadhb, Sec24c, Arid5b, Scn8a, Cacna1c, Mdm4, Rb1cc1, Slc25a25, Cntln, Fbxw7, Naa15, Tmem33, Tmem86a, Ctnnd1, Map3k3, Dpy19l1, Slitrk5, Gpr158, Zfp286a, Abi1, Tph2, Sgcb, Braf, Txk, Pof1b, Fv1, B3galt2, Enc1, Cdk14, Tbc1d9b, Nfatc2, Plekhj1, Ezh1, Hr, Ctdsp1, Fbln5
>rno-miR-217-5p	UACUGCAUCAGGAACUGACUGG	75	0.20	−1.02	Acer3, Zfp711, Usp6nl, Ppm1d, Atp1b1, Med17, Bai3, RGD1566359, Thoc2, Ezh2, Hivep3, Slc38a2, Ubl3, Kctd9, Dennd6a, Anln, Extl2, Stt3a, Yipf6, Tmem47, Fn1, Yaf2, Ythdc1, RGD1307830, Appbp2, Gpm6a, Bicd1, Atp11c, Tm9sf3, Zfyve20, Zbtb5, Morf4l2, Tet2, Ptpn21, Foxo1, Kcnh5, Klhl29, Wdr48, Ube4a, Hnrnpa3, Tmed10, Esco1, Slc45a2, Wapal, Ehmt1, Kctd5, RGD1311595, Myef2
>rno-miR-34c-5p	AGGCAGUGUAGUUAGCUGAUUGC	−	0.62	−	Ttc19, Vamp2, Notch1, Rras, E2f5, Tmem79, Arhgap1, Fam76a, Osgin2, Zfhx4, Ctnnd2, Frk, Soga1, Ap1s2, Fam167a, Ubp1, Camta1, Ppp1r11, Gmfb, Slc44a2, Ptpn4, Svop, Mycn, Zfp644, Map1a, Baalc, Pogz, Eml5, Tbl1xr1, Pitpnc1, Shkbp1, Dgkz, Taf5, Ranbp10, Myrf, Pip5k1a, Akap6, Slc35g2, Asic2, Strn3, Zer1, Daam1
>rno-miR-129-5p	CUUUUUGCGGUCUGGGCUUGC	88	<0.1	−1.20	Prkcb, Rbms3, Cib2, Zbtb20, Zfp281, Hmg1l1, Ube2k, Sc5d, Ctdspl2, Rai14, Ccdc43, Eif4g3, Kctd4, Ago3, Mdm4, Sun2, Rims2, Ythdf1, Tnpo1, Ap3b1, Fam63b, Sacm1l, Pdp2, RGD1559786, Ube4b, Klhl32, Nr2c2, Zfp36l1, Gapt, Wdr61, Duoxa1, Ets1, Pou3f1, Tmub2, Ptp4a1, Nrg2, RGD1307621, Paqr5, Add3, Kctd1

The 3′UTR of Nurr1 and miRNA binding sites were cloned to construct firefly luciferase reporter plasmids (pMIR-REPORT). Plasmids were cotransfected independently with the respective anti- or mimic miRNAs into HEK293T cells. Results showed that the miR-145-5p mimic clearly decreased the luminescence reporter signal to a greater extent (*P* < 0.001; Figure 2D), as did miR-132 and miR-34c-5p, which were previously shown to directly target Nurr1. However, miR-217 exhibited a lower significant interaction when compared with miR-145-5p and miR-34c-5p (Figure 2D). Mutations of the miR-145-5p and Nurr1 recognition sites in the 3′UTR abolished their interaction (Figure 2E). These results suggest that miR-145-5p modulates Nurr1 through its 3′UTR.

### miR-145-5p Regulates Endogenous Nurr1 Levels in Microglia

**Figure 3:**
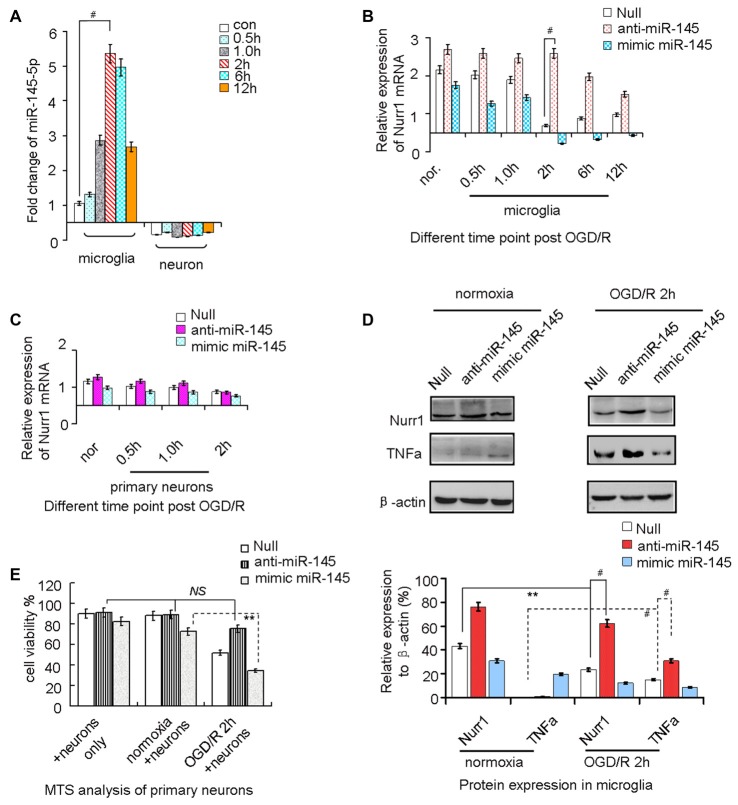
miR-145-5p regulates endogenous Nurr1 levels in microglia. Microglia cultured in normoxia (nor.) and un-transfected (the null group) served as negative controls. **(A)** miR-145-5p expression increased from the onset of OGD/R and peaked at 2 h in microglia (*p* < 0.001), but did not change significantly in primary neurons. **(B)** Transfection anti-miR-145-5p induced an sharply increasing of Nurr1 mRNA post OGD/R 2 h in microglia (*p* < 0.001). **(C)** No obvious changes of Nurr1 mRNA were observed in primary neurons subjected to OGD/R, independent of anti-miR-145-5p or miR-145-5p mimic administration. **(D)** By Western blot assay, Nurr1 protein decreased and TNF-α increased significantly after OGD/R 2 h in microglia. miR-145-5p mimic administration significantly reduced Nurr1 protein level after OGD/R 2 h. **(E)** By MTS analysis of neurons, cell viability of primary neurons cocultured with microglia OGD/R 2 h with anti-miR-145-5p transfection did not show significant increase of cell death when compared to microglia normoxia group or neurons only group (NS: non-significance; *p* > 0.05). Oppositely, administration of miR-145-5p mimic increases neurons death notably in microglia after OGD/R 2 h than those co-cultured with microglia control (*p* < 0.01). **p < 0.01, ^#^*p* < 0.001.

miR-145-5p expression increased from the onset of OGD/R, peaked at 2 h, and then progressively decreased until 12 h in microglia (Figure 3A). However, no significant changes of miR-145-5p expression were observed at different time points of OGD/R in primary neurons (Figure 3A). Nurr1 mRNA levels were reduced from the onset of OGD/R and peaked at 2 h in microglia (Figure 3B). Administration of anti-miR-145-5p induced a sharp increase of Nurr1 mRNA levels in comparison with the null group at each time point of OGD/R in microglia (Figure 3B) but not in primary neurons (Figure 3C).

After OGD/R 2 h induction of miR-145-5p overexpression in microglia, the Nurr1 protein was clearly reduced when compared with cells in the normoxia group (Figure 3D). In particular, expression of TNF-α, which was reported to be transrepressed by Nurr1, displayed a pattern of expression opposite that of Nurr1 under treatment with the miR-145-5p mimic or anti-miR-145-5p (Figure 3D). Cell viability assessed by MTS analysis showed that primary neuronal cells co-cultured with microglia transfected with anti-miR-145-5p post OGD/R 2 h did not exhibit significant cell death when compared with the normoxia group (Figure 3E). Conversely, a noticeable increase in neuronal cell death was observed in those co-cultured with miR-145-5p mimic-transfected microglia (Figure 3E). These experiments suggest that enhanced levels of miR-145-5p inhibit neuronal viability by regulating expression of Nurr1 and TNF-α in microglia after OGD/R.

### Overexpression of Nurr1 Suppresses TNF-α Expression

**Figure 4:**
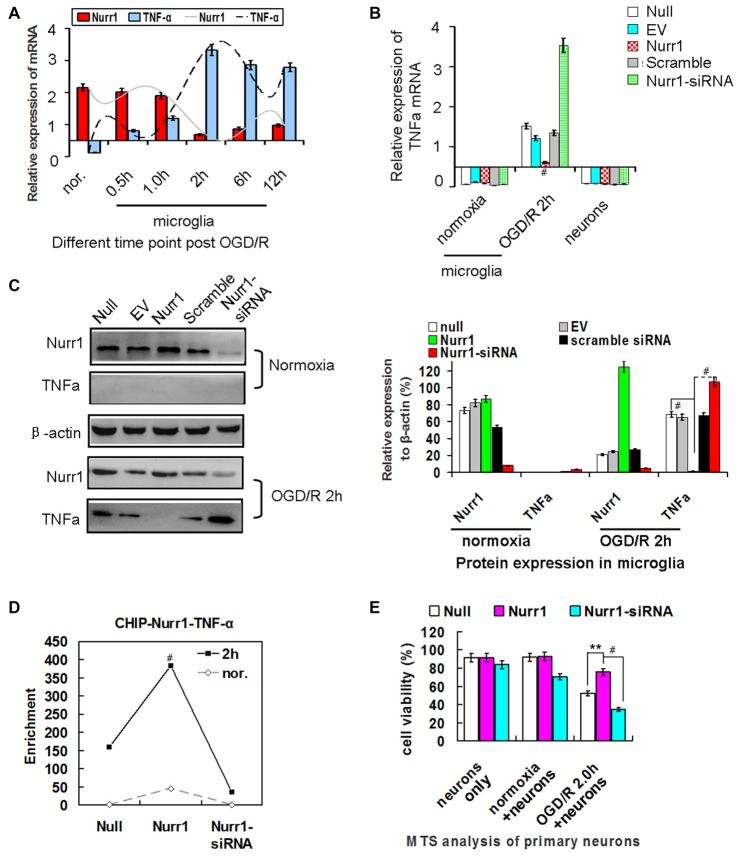
Nurr1 overexpression suppresses TNF-α expression in microglia. **(A)** Quantitation of Nurr1 and TNF-α mRNA in primary microglia which were subjected to OGD/R 0.5 h, 1.0 h, 2 h, 6.0 h and 12 h, respectively. TNF-α mRNA increased from the onset of OGD/R and peaked at OGD/R 2 h in microglia (*p* < 0.001). However, Nurr1 mRNA showed an opposite trend at different time point of OGD/R. **(B)** Overexpression of Nurr1 led to significant attenuation of TNF-α mRNA, otherwise Nurr1-siRNA led to sharply increase of TNF-α expression after OGD/R 2 h in microglia. However, these significant changes of TNF-α mRNA expression were not observed in microglia with normooxia culturing (microglia nor. group) or neurons only group. **(C)** After OGD/R 2 h in microglia, Nurr1 overexpression by pcDNA-Nurr1 transfection significantly inhibited TNF-α protein expression (*p* < 0.001). Oppositely, Nurr1 knockdown by siRNA interference significantly restored TNF-α protein expression (*p* < 0.001). **(D)** *ChIP* assay of Nurr1 on the TNF-α promoter in response to OGD/R 2 h in microglia cells. Nurr1 overexpression by plasmid transfection increased Nurr1 occupancy at the TNF-α promoter, especially when TNF-α expression reached great amount post OGD/R 2 h. Data are displayed as fold enrichment over control IgG. **(E)** By MTS analysis of neurons, neurons viability co-cultured with microglia OGD/R 2 h which were transfected with Nurr1 overexpression plasmid showed significant lower ratio of cell death than the null (*p* < 0.01) and Nurr1-siRNA group (*p* < 0.001). However, no obvious changes of cell viability were observed in groups of neurons only and neurons+microglia normoxia after overexpression or knockdown Nurr1 expression. **p < 0.01, ^#^*p* < 0.001.

It was previously reported that Nurr1 induces transrepression of TNF-α by binding to its promoter in Parkinson’s disease (Saijo et al., 2009). Herein, we sought to determine the effects of aberrant expression of Nurr1 on TNF-α expression in microglia and primary neurons subjected to OGD/R at different time. Results of qRT-PCR analysis showed that TNF-α mRNA expression increased and peaked at 2 h of OGD/R in microglia which is opposite to Nurr1 mRNA levels (Figure 4A), but not in primary neurons (data not shown). Upon treatment with the Nurr1 activation plasmid, overexpression of Nurr1 significantly attenuated TNF-α mRNA and protein expression. Conversely, an increase in TNF-α expression was observed after treatment with Nurr1-siRNA after OGD/R 2 h in microglia (Figures 4B,C). However, no significant changes were observed in primary neurons (Figure 4B).

To assess whether Nurr1 binds to the *TNF-α* gene promoter, we performed a chromatin immunoprecipitation (ChIP) assay in microglia. As shown in Figure 4D, Nurr1 plasmid transfection increased Nurr1 occupancy at the TNF-α promoter, especially when TNF-α expression reached a high level after OGD/R 2 h. Furthermore, neuronal cells co-cultured with microglia after OGD/R 2 h with Nurr1-siRNA transfection showed significant cell death and could be rescued by Nurr1 overexpression after transfection with the Nurr1 plasmid (Figure 4E). In contrast, knockdown or overexpression of Nurr1 did not alter cell viability independent of OGD/R in the primary neuron culture only group (Figure 4E). These data demonstrate that lower expression of Nurr1 relieves transrepression of TNF-α in microglia and inhibits cell growth in primary neurons after OGD/R 2 h.

### Abnormal Inactivation of Nurr1-Mediated Transrepression on TNF-α by miR-145-5p Overexpression Accelerates Inflammatory Injury in Acute MCAO/R of Rats

**Figure 5:**
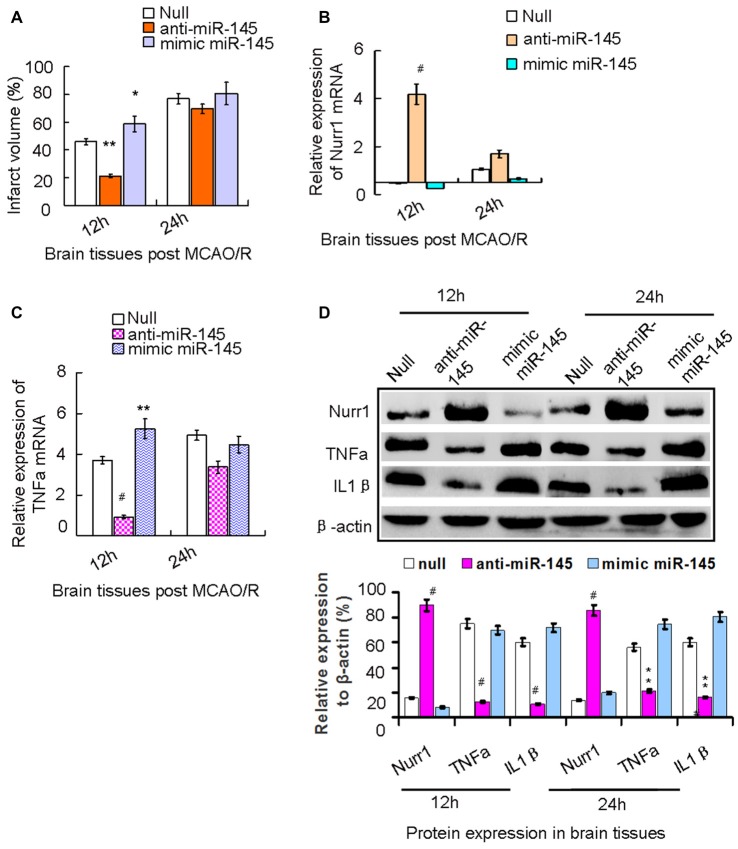
The axis signaling of miR-145-5p-Nurr1-TNF-α in acute *MCAO/R* model of rats by *in vivo* expriments. Infarct volume was plotted as the percentage of ipsilateral cerebral. **(A)** After 12 h of MCAO/R, administration of anti-miR-145-5p *in vivo* via ICV injection to ischemic rats immediately reduced Infarct volume by 39.05% (*p* < 0.01). While administration of miR-145-5p mimic increased infarct volume by 15.05% when compared to the null group (*p* < 0.05). However, these changes of infarct volume were not observed significantly at 24 h. **(B)** By RT-qPCR analysis, Nurr1 mRNA expression significantly increased in anti-miR-145-5p injection samples (*p* < 0.001) whereas decreased after administration of mimic miR-145-5p (*p* < 0.05). **(C)** In contrast, TNF-α mRNA expression significantly decreased in anti-miR-145-5p injection samples post 12 h of MCAO/R (*p* < 0.001) whereas significantly increased when administration of miR-145-5p mimic (*p* < 0.01). However, these significant changes of TNF-α mRNA expression were not observed in samples post 24 h of MCAO/R. **(D)** By western blot analysis, both TNF-α and IL1β expression levels were significantly suppressed by Nurr1 overexpression with administration of anti-miR-145-5p at 12 h and 24 h post-MCAO/R, and vice versa. *p < 0.05, **p < 0.01, ^#^*p* < 0.001.

We demonstrated that increasing expression of miR-145-5p peaked at 12 h in our rat MCAO/R model (Figure 2A). We next administered the miR-145-5p mimic and anti-miR-145-5p *in vivo* via ICV injection into ischemic rats immediately after MCAO. Mean infarct volumes were measured at 12 h and 24 h post-MCAO/R. Administration of anti-miR-145-5p reduced infarct volume by 24.05% at 12 h, whereas administration of the miR-145-5p mimic increased infarct volume by 11.05% (Figure 5A). Administration of anti-miR-145-5p significantly increased Nurr1 mRNA expression (Figure 5B) and reduced TNF-α mRNA expression at 12 h post-MCAO/R (Figure 5C) but not at 24 h post-MCAO/R.

**Figure 6:**
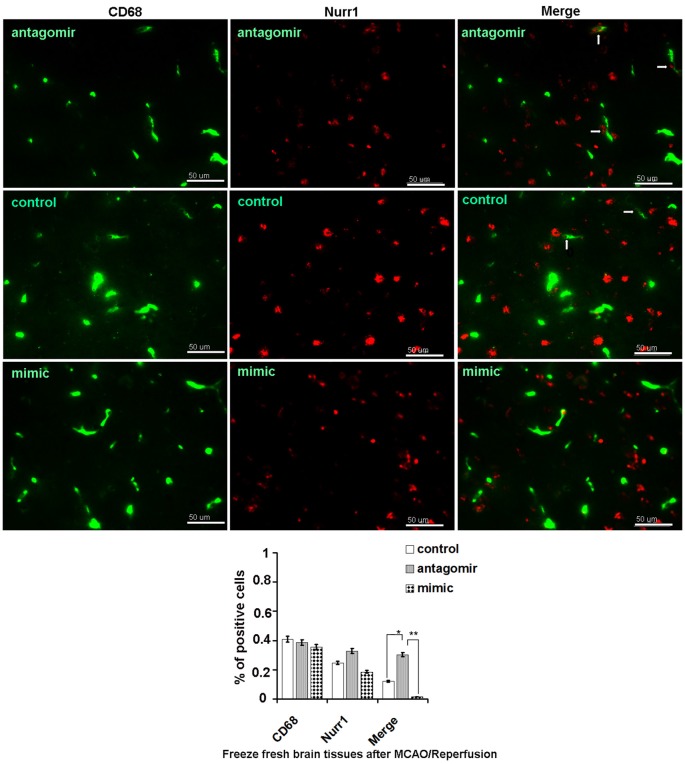
Nurr1 expression in active microglia with administration of miR-145-5p in peri-infarct areas post 12 h of MCAO/R. Double immunofluorescence staining shows more expression of Nurr1 in active microglia with administration of anti-miR-145-5p, and little expression of Nurr1 with administration of miR-145-5p mimic in the peri-infarct areas bordering with intact tissues post 12 h of MCAO/R. Scale bars = 50 μm. Arrows indicate co-localization of CD68 and Nurr1 in active microglia. *p < 0.05, **p < 0.01.

Accordingly, Nurr1 protein expression increased 4.53- and 3.42-fold upon treatment with anti-miR-145-5p at 12 h and 24 h post-MCAO/R, respectively (Figure 5D). Both TNF-α and IL-1β protein levels were significantly up-regulated at both 12 h and 24 h post-MCAO/R, and they were successfully suppressed by Nurr1 overexpression induced by administration of anti-miR-145-5p (Figure 5D). Accordingly, Nurr1 expression increased significantly upon anti-miR-145-5p treatment vs. miR-145-5p mimic treatment in active microglia in peri-infarct areas post 12 h of MCAO/R using immunofluorescence (Figure 6). These data reveal that lower expression of Nurr1 induced by miR-145-5p upregulation increases infarct volume at an early stage of cerebral ischemia of rats by activating TNF-α and IL-1β proinflammatory signals.

### miR-145-5p Interruption Facilitates Neurological Outcome of Rats Post MCAO/R

**Figure 7:**
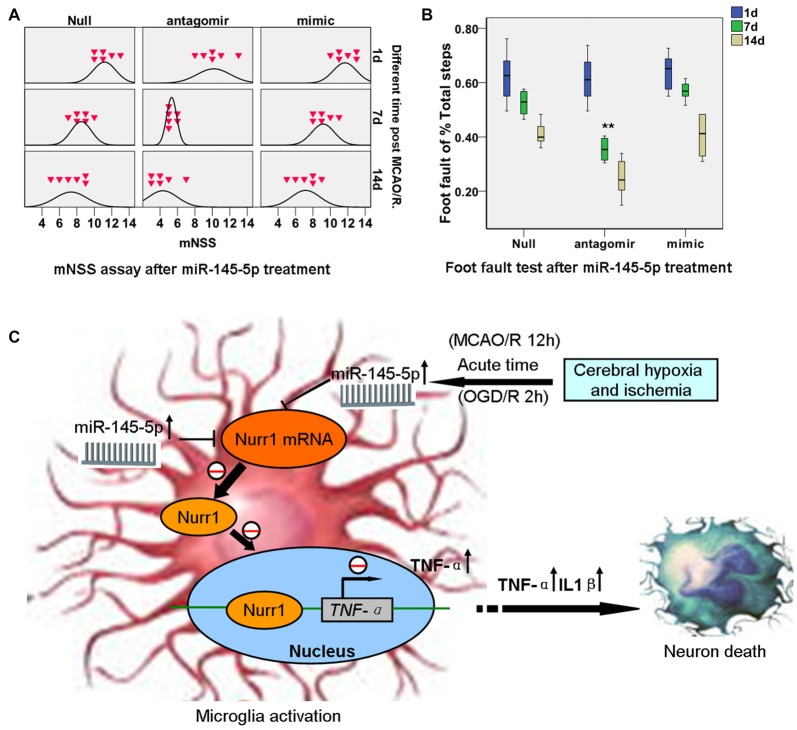
miR-145-5p interruption facilitates neurological outcome of rats post MCAO/R. Modified Neurological Severity Score (mNSS) and foot fault tests were assessed on days 1, 7 and 14 after MCAO/R. **(A)** mNSS in antago-miR-145-5p animals were significantly decreased at both 7d and 14d after MCAO/R (*p* < 0.05). **(B)** Foot fault test, for the front left and hind left limbs were significantly lower in antagomiR-145-5p animals than that of miR-145-5p mimic and null animals. *n* = 54 for the combination group. **(C)** The graphical summary. Here, we describe a novel miR-145-5p regulatory mechanism of Nurr1 that can act downstream of TNF-α activation. In acute cerebral ischemia (MCAO/R 12 h) of rats and OGD/R 2 h of microglia, Nurr1 inhibits TNF-α expression by binding promoter of *TNF-α* gene. This regulatory effect is inhibited by Nurr1 protein decline that induced by miR-145-5p overexpression. Blocking the abnormal activation of miR-145-5p-Nurr1-TNF-α axis signaling can relieve neurons death upon MCAO/R of rats in acute time. **p < 0.01.

Significant functional deficits were observed in the mNSS for animals subjected to mimic miR-145-5p administration compared to anti-miR-145-5p animals at both 7d and 14d after injury (*p* < 0.05; Figure 7A). For foot fault tests, the percentages of front left, hind left, total left, and total foot faults at 7d and 14d after injury after anti-miR-145-5p was significantly lower, compared to that of null and mimic miR-145-5p animals (*p* < 0.05; Figure 7B). However, in the mNSS and foot fault tests were not significantly different between the null and miR-145-5p mimic administration animals (*p* > 0.05).

## Discussion

The present study utilized a coculture model system in which neurotoxicity was induced by activated microglia post OGD/R 2 h. We showed several lines of evidence that Nurr1 plays an important role in protecting neurons from inflammation-induced neurotoxicity by inhibiting expression of inflammatory genes in microglia. Firstly, clear reductions in both Nurr1 mRNA and protein expression were detected in microglia post OGD/R 2 h and accompanied by a substantial elevation in TNF-α gene expression (Figures 4A,C). Furthermore, Nurr1 overexpression induced by Nurr1 plasmid indeed leaded to inhibition of TNF-α mRNA and protein expression in microglia post OGD/R 2 h (Figures 4B,C). Secondly, reduction of Nurr1 expression induced by OGD/R 2 h in microglia resulted in increased neuronal cell death that could be rescued by Nurr1 plasmid transfection (Figure 4E). Thirdly, ChIP assay demonstrated that Nurr1 was recruited to the TNF-α promoter following OGD/R 2 h treatment and was further enhanced by Nurr1 overexpression (Figure 4D), indicating that it was acting locally to repress transcription. This finding is consistent with previous studies showing that reduced Nurr1 expression results in death of tyrosine hydroxylase-expressing neurons in Parkinson’s disease by targeting the TNF-α gene promoter in microglia (Saijo et al., 2009; Kim et al., 2015). Finally, Nurr1 declined sharply in brain tissues at 12 h following MCAO/R, and lower Nurr1 expression was partly correlated with infarct volume 12–24 h post MCAO/R by *in vivo* assays (Figure 2A).

Results of our study strongly indicate transcript factor Nurr1 protects neurons from ischemia-induced inflammation injury, at least in part, through transrepression of TNF-α in activated microglia post OGD/R. These are supported by several recent studies: (1) up-regulation of VEGF and Nurr1 strongly promote DAergic neuronal survival after Cystatin C treatment, which might be involved in p-PKC-α/p-ERK1/2-Nurr1 signaling and autophagy (Zou et al., 2017). (2) The interaction between Nurr1 and Foxa2 protects midbrain dopamine neurons against various toxic insults, while their expression are absent during aging and degenerative processes (Oh et al., 2015). (3) The Glial cell line-derived neurotrophic factor (GDNF) was found to protect neurons in cerebral ischemia, and Nurr1 was up-regulated by GDNF in the process of dendritic and electrical maturation of neuron cells (Cortés et al., 2017).

The important roles of changes in miRNA expression in ischemic brain injury have been discovered recently using miRNA profiling techniques in a rat MCAO/R model (Dharap et al., 2009; Di et al., 2014). In the present study, miR-145-5p mimic clearly decreased Nurr1 expression in HEK293T (*P* < 0.001), as did miR-132 and miR-34c-5p, which was consistent with the previous study that miR-34c-5p directly regulated Nurr1 in HCT116 cells (Beard et al., 2016) and miR-132 targeted Nurr1 in differentiation of dopamine neurons (Yang et al., 2012). However, miR-217 exhibited a lower significant interaction when compared with miR-145-5p and miR-34c-5p (Figure 2D). Further mutation of the miR-145-5p and Nurr1 recognition sites in the 3′UTR abolished their interaction in HEK293T cells as detected by luciferase reporter assay (Figure 2E). However, Nurr1 expression is not regulated by miR-365-3p. These data demonstrate that it was essential to elucidate the specific relation between miR-145-5p and Nurr1 in rat model of cerebral I/R injury.

miR-145-5p has been shown to be up-regulated in the pathological process of vascular neointimal lesion formation (Saugstad, 2010; Li et al., 2012), cardiomyocyte survival (Chen et al., 2015), and H_2_O_2_-induced neuronal injury (Gan et al., 2012). Here, we observed that miR-145-5p expression sharply increased in the cortex of rats 12 h post MCAO/R (Figure 2B) and in isolated primary microglia post OGD/R 2 h (Figure 3A). Modulation of miR-145-5p expression affects cell viability under *in vitro* ischemic conditions, and this occurs via regulation of Nurr1 (Figures 3D,E). Administration of miR-145-5p mimic in primary microglia post OGD/R significantly reduced TNF-α expression and IL-1β by enhancing Nurr1 expression, which subsequently improved neuronal cell viability (Figures 3E, 4E). As expected, anti-miR-145-5p administration via ICV injection reduced infarct volume by 24.05% at 12 h post MCAO/R in rats (Figure 5A). Whatever, more researches must be performed to elucidate the specific effects of miR-145-5p on cerebral I/R injury in human blood or cerebrospinal fluid.

CD68 is specifically expressed in microglia in ischemic stroke brain (Szalay et al., 2016). By immunofluorescence assay, massive Nurr1 expression was observed in active microglia after anti-miR-145-5p treatment (Figure 6). Our findings are in agreement with a previous report, which demonstrated that antagomir-145 infusion resulted in a decreased area of infarction at 1 day of reperfusion by increasing SOD2 protein expression (Gan et al., 2012). It is noteworthy that 24 h after MCAO/R in rats, miR-145-5p inhibition could not reduce infarct volume despite the significant increase in Nurr1 (Figure 5A). Furthermore, significant functional improvement were observed in the mNSS and foot fault tests for animals subjected to mimic miR-145-5p administration compared to anti-miR-145-5p animals after cerebral I/R injury (Figures 7A,B). This indicates that the supression of miR-145-5p, which decreased neuron cell death by increasing TNF-α-mediated inflammation, might be insufficient to provide protective effects under these conditions. Therefore, it is still worth exploring function and mechanism(s) of miR-145-5p/Nurr1/TNF-α Signaling in human stoke in the near future.

## Conclusion

In summary, our study identified and confirmed a novel regulation of Nurr1 by miR-145-5p in both *in vitro* and *in vivo* cerebral ischemic conditions. The hypothetic mechanisms were examined in isolated primary microglia and neurons post OGD/R by *in vitro* study (Figure 7C). I/R-induced miR-145-5p overexpression suppresses Nurr1 protein expression and attenuates Nurr1 transrepression of the *TNF-α* promoter in microglia (OGD/R 2 h), which then causes TNF-α-related neuronal injury. Additional *in vivo* studies have shown that administration of anti-miR-145-5p could increase Nurr1 expression and reduce subsequent infarct volume in acute cerebral ischemia (MCAO/R 12 h). It may be an effective therapeutic strategy of reducing neuronal injury following MCAO/R in rats by blocking abnormal activation of miR-145-5p/Nurr1/TNF-α axis signaling in the acute phase.

## Author Contributions

XX and YoZ conceived and designed the experiments. XX, LP and YaZ conducted the experiments. XX and LL analyzed the results. LP, YC, JZ, SY and XX contributed materials and analysis tools. XX wrote the article. YoZ is the corresponding author. All authors reviewed the manuscript.

## Conflict of Interest Statement

The authors declare that the research was conducted in the absence of any commercial or financial relationships that could be construed as a potential conflict of interest.
